# Change people attitudes towards schizophrenia using a short video

**DOI:** 10.1192/j.eurpsy.2022.322

**Published:** 2022-09-01

**Authors:** D. Amsalem

**Affiliations:** Columbia University, NYC, NY, USA, Psychiatry, NYC, United States of America

**Keywords:** intervention, schizophrénia, stigma, RCT

## Abstract

**Introduction:**

Social contact-based video interventions effectively reduce stigma toward individuals with psychosis.

**Objectives:**

We recently demonstrated the efficacy of a 90-second social contact–based video intervention in reducing stigma. The current randomized controlled study presents four briefer videos differing in presenter’s gender and race, with baseline, postintervention, and 30-day follow-up assessments. The study aimed to examine whether people changing their attitudes following the intervention.

**Methods:**

Using a crowdsourcing platform (CloudResearch), we recruited and assigned 1,993 race and gender-balanced participants ages 18–35 years to one of four brief video-based interventions (Black female, White female, Black male, and White male presenters) or a nonintervention control condition. In the videos, a young presenter with psychosis humanized their illness through an evocative description of living a meaningful and productive life.

**Results:**

Five-by-three ANOVA showed a significant group-by-time interaction for the total score of all five stigma domains: social distance, stereotyping, separateness, social restriction, and perceived recovery. A one-way ANOVA showed greater reductions in video intervention groups than control at post-intervention and 30-day follow-up, but no differences between video groups.

**Conclusions:**

This randomized controlled study replicated and extended previous research findings by showing stigma reduction across videos that differ in the presenter’s gender and race, thus enhancing generalizability. The videos described the experience of psychosis and reduced stigma, suggesting their potential utility on social media platforms to increase the likelihood of seeking services and ultimately may improve access to care among young individuals with psychosis. Future research should address intersectional stigma experienced by culturally tailoring the narrative.

**Disclosure:**

No significant relationships.

